# Machine learning informed additive manufacturing of stainless steel 410 using cold metal transfer-based metal inert gas welding

**DOI:** 10.1007/s00170-026-18086-6

**Published:** 2026-04-18

**Authors:** Swati Singh, Amritbir Singh, Shiva Sekar, Rajab Alsayegh, Shrikrishna Nandkishor Joshi, Saurav Goel

**Affiliations:** 1 Department of Mechanical Engineering, Indian Institute of Technology Guwahati, Guwahati, 781039 India; 2 Laboratory for Advanced Manufacturing and Processing, Indian Institute of Technology Jammu, Jammu, 181221 India; 3 Department of Mechanical Engineering, Faculty of Engineering, King Abdulaziz University, P.O. Box 80200, Jeddah, 21589 Saudi Arabia; 4 School of Engineering and Design, London South Bank University, 103 Borough Road, London, SE1 0AA United Kingdom; 5 School of Advanced Engineering, University of Petroleum and Energy Studies, Dehradun, 248007 India; 6 China Beacons of Excellence Research and Innovation Institute, University of Nottingham Ningbo China (UNNC), Ningbo, 315101 China

**Keywords:** Artificial intelligence, Additive manufacturing, regression models, Cat-Boost Regression

## Abstract

This study investigates the use of machine learning to predict bead geometry in cold metal transfer (CMT)–based wire-arc additive manufacturing (WAAM) of SS410 martensitic stainless steel. A dataset comprising 50 experimentally deposited single beads was developed indigenously to model the relationship between key process parameters and the resulting bead aspect ratio. Multiple regression and advanced tree-based ensemble models, including Random Forest, XGBoost, Extra Trees Regressor and Cat-Boost Regressor (CBR), were implemented to capture the influence of wire feed rate, deposition rate (torch travel speed), current and voltage on bead morphology. Comparative evaluation of the models, supported by independent validation demonstrated that the CBR model provides the most accurate prediction of aspect ratio among other models investigated in this study. Feature importance analysis indicated that welding current is the dominant parameter governing bead geometry, followed by voltage. Microstructural characterisation of the thick walls produced using the optimal process parameters revealed a progressive increase in δ-ferrite content with build height, associated with heat accumulation during multilayer deposition. Correspondingly, the ultimate tensile strength and yield strength decrease by 14% and 12.7%, respectively. These findings highlight the potential of machine-learning-based frameworks for predicting and optimising process–geometry relationships in WAAM, while also indicating the need for future AI-assisted strategies to control phase evolution and mitigate the formation of detrimental microstructural constituents in martensitic stainless steels.

**Supplementary Information:**

The online version contains supplementary material available at 10.1007/s00170-026-18086-6.

## Introduction

Among all manufacturing procedures, additive manufacturing (AM) has been widely recognised for its ability to reduce material waste, reduce cost and shorten production lead times [1]. As AM technologies continue to evolve and expand into different material domains, such as plastics, metals and ceramics, wire-arc additive manufacturing (WAAM) has emerged as a promising technique. Known for its high deposition rates, cost-effectiveness in machinery and feedstock, and superior material properties, WAAM has garnered substantial attention within the manufacturing landscape [2]. This technology’s advancements align with the broader trajectory of AM innovation, further establishing WAAM as a leading additive manufacturing methodology.

WAAM is an approach that uses an arc as an initial source of thermal energy to apply successive layers of material [3]. Furthermore, metal wire is an economical option in comparison to metal powder, resulting in lower overall logistics. WAAM typically uses heat inputs ranging from 10 to 100 J/mm. This critical variable affects both the microstructure and the characteristics of the welding bead [4]. During successive layering process, heat accumulation in the component increases with the build height. Hence, in order to manufacture large components, it is important to minimise the level of thermal energy generated during the process. Given the challenges posed by higher heat input in the WAAM process, it becomes imperative to explore effective solutions. In this context, the cold metal transfer (CMT) approach has emerged as a promising strategy to mitigate the adverse effects of excessive heat. CMT uses an innovative wire feed method along with advanced digital control to precisely manage material deposition while minimising the heat input [5].

It is important to examine the integration of ML with the building blocks of WAAM, specifically single bead (single track) deposition and its characteristics. Rosli et al. [6] utilised the response surface methodology (RSM) approach to optimise parameters such as wire feed rate (WFR), travel speed (TS) and pulse. Their optimisation work resulted in the highest possible bead width and height, while minimising roughness. Similarly, Huang et al. [7] used RSM to effectively optimise the input parameters and reduce the incidence of solidification cracking in 4043 Al alloy produced using WAAM. Vora et al. [8] effectively adjusted independent factors to produce faultless thin wall structures using 2.25 Cr-1.0 Mo steel. Their experiments used the Box-Behnken design, which considers WFS, TS and voltage as independent factors, while bead width and height were treated as dependent variables. In addition, several investigations were conducted on different materials to determine the optimal parameters for minimising flaws and ensuring the formability of the part. Recently, Zavdoveev at al. [9] used Taguchi methods to optimise processing parameters of pulsed based WAAM for austenitic SS to achieve better weld formation. Ni et al. [10] used RSM approach to enhance the accuracy of weaving deposition on curved geometries. Their results demonstrated that the aspect ratio is a crucial parameter for defining bead quality and is significantly affected by weaving width and WFS.

Most optimisation approaches currently employed in metal additive manufacturing arena rely on trial-and-error methods. These methods are inherently associated with significant disadvantages, including high time consumption and substantial costs. Additionally, many research articles utilise the RSM approach for parametric optimization, which presents several limitations in contrast to the more advanced and efficient techniques offered by machine learning (ML) [11]. Firstly, RSM relies on pre-established mathematical models, which may limit the exploration of complex data correlations when the true relationships deviate from the assumed polynomial structure [12]. In contrast, ML algorithms can flexibly analyse diversified data, efficiently revealing complicated patterns without the requirement of explicitly defining the functional form in advance. Secondly, RSM typically models the response surface using polynomial functions including linear, quadratic and interaction terms to represent nonlinear relationships between input and output parameters. While this approach is effective for moderately nonlinear systems, its performance may decline when the underlying process exhibits highly complex or non-polynomial relationships that are difficult to approximate with predefined polynomial models. In contrast, ML techniques are known for their ability to capture complex nonlinear relationships [13]. Thirdly, RSM models are susceptible to fundamental assumptions while constructing the model. On the other hand, ML approaches can handle large sets of data distributions more robustly.

Apart from RSM, which relies on predefined polynomial response models, more advanced modelling approaches such as machine learning and physics-based simulations have recently been explored. Consequently, many researchers have investigated the viability of incorporating ML approaches to address these challenges in optimising metal AM processes.

ML concentrates on building algorithms and models that allow learning from input data and making predictions without using programming. It employs statistical approaches to identify correlations within data, enabling the learning through repeated applications and improving the performance with time. This approach incorporates training of the algorithm on input data, wherein iterations are conducted until the errors associated with the predicted variables are minimised [14, 15].

Barik et al. [16] used a support vector regression-based ML model to optimise the Bead Width (BW), Bead height (BH) and tensile strength using current, voltage and TS as process variables. Oh et al. [17] used a similar ML model to remove the irregularities in the WAAM fabricated SS304 single track through optimisation of the BW and BH. Support vector regression has some inherent disadvantages which limit its use for large datasets. Therefore, along with support vector regression, Kunchala et al. [18] used a hybrid-teaching learning-based optimisation (h-TLBO) technique to optimize the weld bead characteristics. Their results showed good agreement of h-TLBO with experimental results and a maximum error margin of 4%.

Zheng et al. [19] developed a forecasted model for BW and BH using particle swarm optimisation based back-propagation neural network algorithm. This optimisation technique leads to the refinement of prediction model and achieved improved accuracy in estimating the BW and BH of duplex stainless-steel part.

Kumar and Maji [20] utilised both RSM and genetic algorithms for appropriate selection of process variables for near-net shape fabrication of SS304L using WAAM. Their study concluded that the selection of optimal bead size and degree of overlap transcends a one-size-fits-all approach. Instead, these parameters should be meticulously calibrated based on the intricacies of the desired final form. Rao et al. [21] utilised artificial neural networks (ANN) and finite element methods (FEM) to predict the BH and BW of WAAM fabricated single tracks of mild steel. Their findings suggested that the FEM approach necessitates multiple steps and requires considerable time to predict response variables, but the ANN method was deemed well-suited to address these challenges. Qin et al. [22] utilized a long short-term memory neural network-based ML model and reported this approach to be effective in improving the process stability, consequently enhancing the quality of the Ti6Al4V part. Drawing from the literature, investigators have extensively applied ML based models to various materials such as SS308 [23], Ti6Al4V [24], SS316L [25] etc. These studies exhibit the potency of ML models in optimising the welding variables to enhance the quality of a WAAM fabricated product [26–28].

Nevertheless, despite its broader application, a significant gap remains in the literature regarding the use of ML approaches particularly to optimise the SS410 bead. This presents an opportunity for further exploration, as addressing this research gap could offer novel insights into the optimisation of WAAM fabricated SS410 material. SS410 is classified as a martensitic stainless steel and is used in several industries such as petroleum, gas, manufacturing, turbines and defense for various applications.

While machine learning approaches have been explored for several alloys used in wire arc additive manufacturing, including austenitic stainless steels such as SS316L, their application to martensitic stainless steels such as SS410 remains limited. The distinct phase transformation behaviour and microstructural evolution associated with SS410 during WAAM necessitate dedicated investigation of the process–geometry–property relationships for this material system. The application of ML to WAAM-fabricated SS410 provides a predictive framework for understanding the relationship between process parameters and resulting bead geometry. To the best of the authors’ knowledge, systematic machine-learning-based modelling of bead geometry for WAAM-fabricated SS410 has not been reported in the open literature.

Extra Tree Regressor (ETR), XGBoost (XGB), and Cat-Boost Regressor (CBR) were applied to the single bead (single track) experiments of SS410 to predict the bead aspect ratio. In the modelling framework, wire feed rate, deposition rate (torch travel speed), welding current and voltage were treated as input predictor variables, while the aspect ratio of the deposited bead was considered the response (target) variable. In particular, tree-based ensemble algorithms were selected because they are well suited for experimental manufacturing datasets, as they can capture nonlinear parameter interactions, handle correlated predictors and maintain robust predictive performance even with relatively limited experimental data.

The remainder of this manuscript is organized as follows. Section 2 describes the experimental setup, materials and data acquisition procedure used in the CMT-based WAAM process, as well as the development of the machine learning models and their predictive performance in estimating the bead aspect ratio. Section 3 provides a comparative analysis of different regression models and evaluates their predictive accuracy. Section 4 discusses the microstructural evolution and mechanical properties of the fabricated thick walls. Finally, Sect. 5 summarises the key findings and conclusions of the present study.

## Experimental details and analysis

**Fig. 1:**
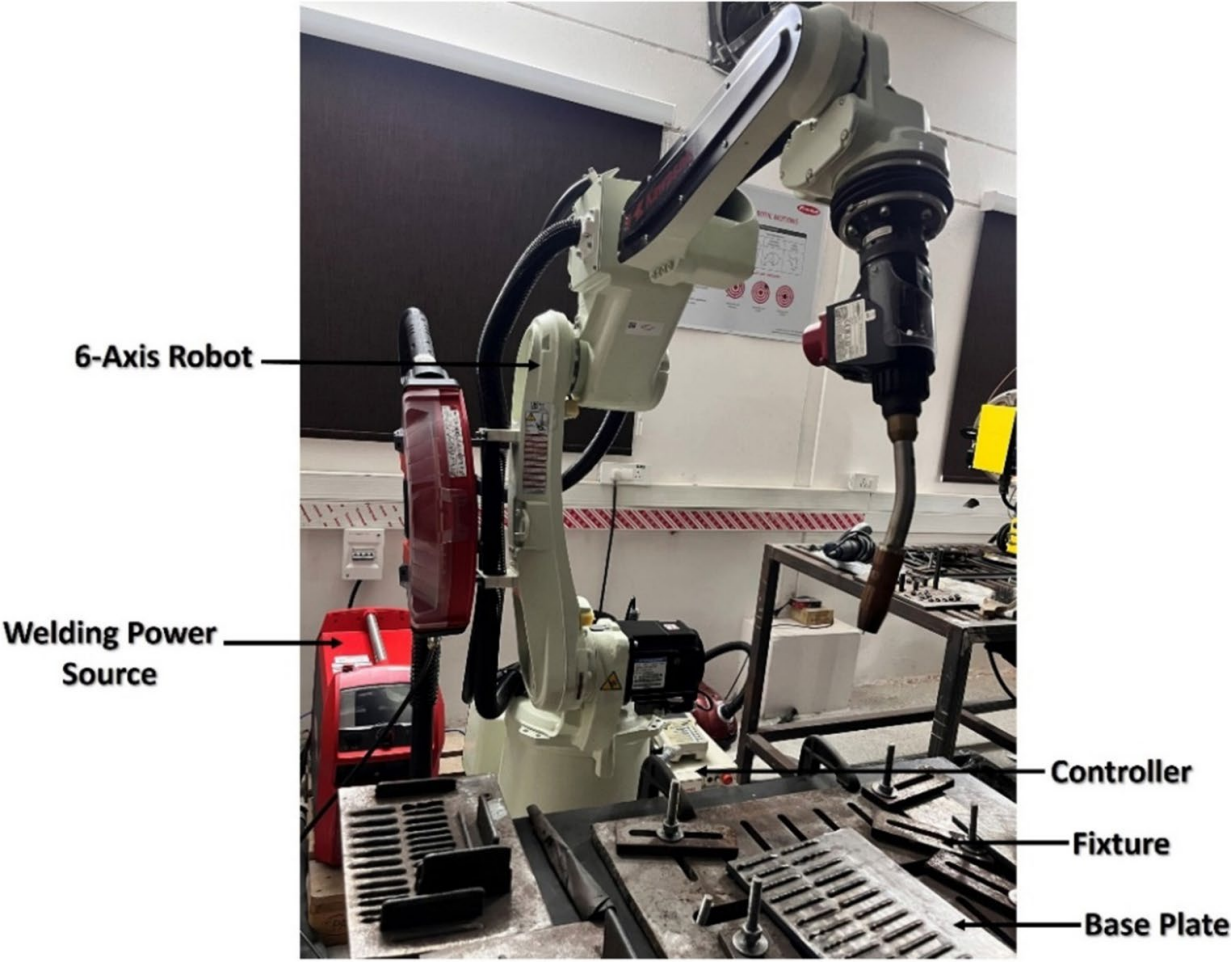
The CMT based welding process used to deposit single track beads of SS410

A commercially available 1.2 mm diameter SS410 wire was used for the deposition process. The chemical composition of SS410 feedstock and the mild steel base plate are shown in Table 1. This wire was applied onto mild steel plates measuring 200 mm × 300 mm × 10 mm. Prior to welding, the base plate underwent finishing and was then degreased with acetone to remove any contaminants on the substrate that could potentially impair the fabrication process. The experiments were performed using the CMT-based MIG welding technique with the TPS400I setup from Fronius, Austria. The deposition was controlled by a 6-axis robot (Kawasaki, 3BA0061N, Japan) following pre-defined trajectories as shown in Fig. 1. In addition, a mixture of 98% argon and 2% oxygen was utilised for shielding during the deposition process.

**Table 1:** The composition of SS410 wire and the base plate for single-track and bulk deposition in the WAAM process

Composition	C	Ni	Mn	Si	*P*	S	Cu	Cr	Mo	Fe
Feedstock wire (SS410)	0.08	0.29	0.56	0.4	0.019	0.01	0.16	12.2	0.09	bal
Base plate (MS)	0.05	-	0.7	0.12	0.016	0.004	-	-	-	bal

**Fig. 2:**
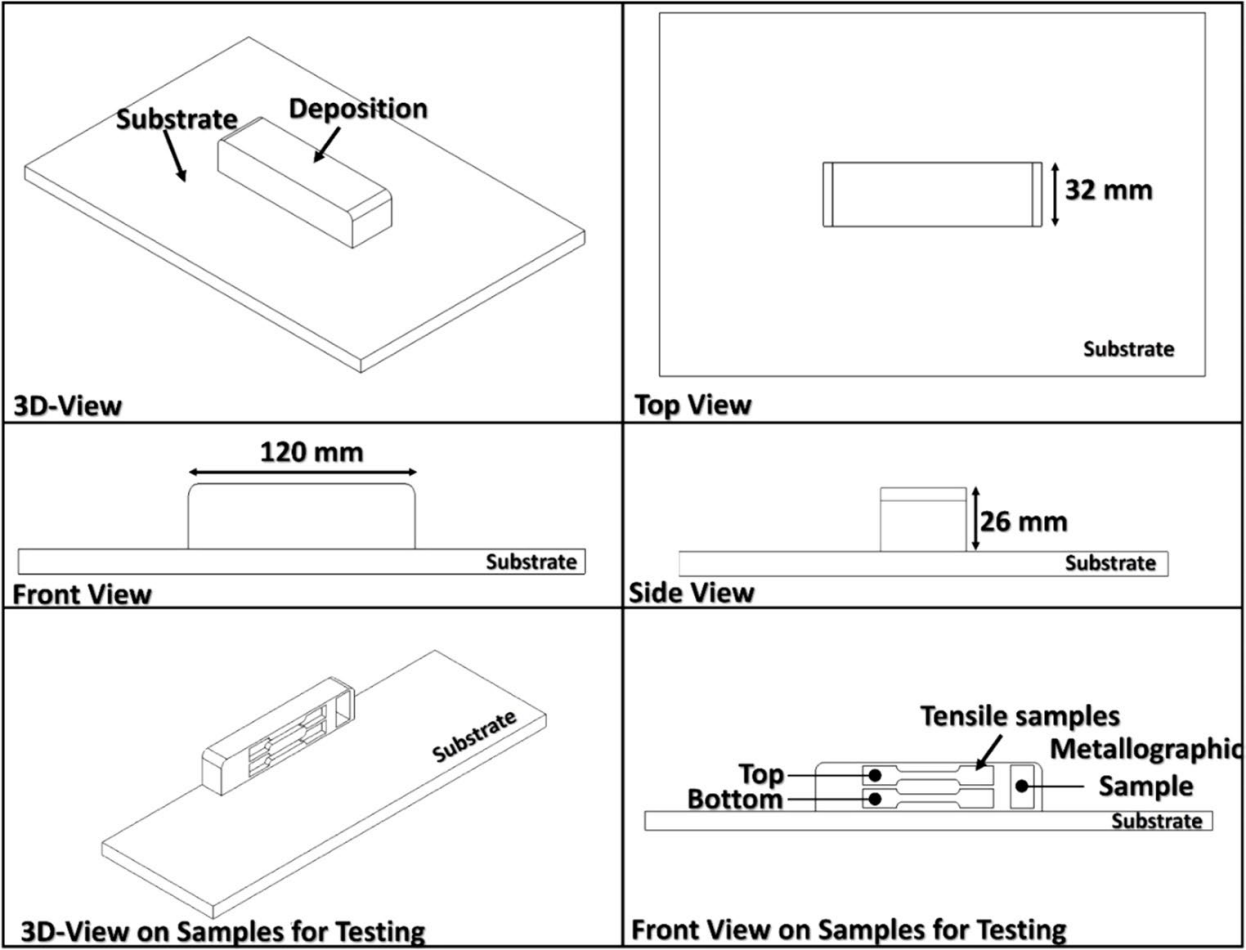
The schematic illustration of the design and development of a thick wall, aimed at investigating its mechanical properties and microstructural analysis

In the subsequent section, we detail the construction of thick walls at optimum parameters. This process was extended across a total of 12 layers, each layer consisting of 8 different beads overlapped to form overall dimensions of 120, 32 and 26 mm of length, width and height, respectively. The contact tip to work distance (CTWD) of the welding torch nozzle was maintained at 12 mm from the substrate. The thick wall was built with an inter-layer time of 60 s and an inter-bead time of 120 s. Following this, two tensile samples were prepared from different locations relative to the building height and were labeled as “bottom” and “top” samples. Additionally, a sample for materials characterization was sectioned as depicted in Fig. 2. A mixture consisting of 98% Ar and 2% O_2_ was used to provide shielding throughout the entire procedure of deposition.

Subsequently, the specimens were extracted from the thick wall to conduct characterisation analysis. For sample preparation, emery paper ranging from 320 to 2500 grit was employed, followed by diamond polishing. This multi-step process ensured a high-quality surface finish, essential for accurate analysis and characterization. For microstructural analysis, the specimens were exposed to the kalling’s reagent. The microstructure was studied by means of optical microscope, specifically the LEICA DM2700 M, and a scanning electron microscope, namely the Jeol JSM-6610LV. X-ray diffraction analysis was performed utilizing a Malvern Panalytical Empyrean instrument to characterise various phases, with in a range of scan angle between 20 and 90 degrees with a step size of 0.01 degrees.

The lateral dog-bone samples were prepared from different positions of the thick wall to fulfill the subsize criteria of ASTM E8 for tensile examination. The tensile examination was carried out employing a universal tensile testing equipment of Walter + bai, LFV, Switzerland with an input load rate of 1.5 kN/min. Additionally, the density of the specimens was determined using Archimedes principle. The hardness of the specimens was evaluated with Vickers microhardness testing equipment, specifically the Inova Test Falcon 400 from the Netherlands.

### Experimental data

In order to obtain desirable closely packed highly dense, crack-free multiple layer alignment (without staircase effect), while avoiding interfacial layer diminishing, and maintaining proper meniscus shape, we conducted 50 runs of CMT-based WAAM, using various combinations of wire feed rate (f) in mm/min, deposition rate (d) in mm/min, current (I) in Ampere, and voltage (V) in Volts. The results of aspect ratio for various combinations of these four crucial parameters are presented in Table 2. The best value of aspect ratio of 3.46 was observed for a WFR of 5600 mm/min, deposition rate of 750 mm/min, current of 190 amperes and voltage of 16.4 volts. This ensures the deposition of a hump-free thick wall.

**Table 2:** The experimental design and the data collected from each run of the CMT-based WAAM process

RUNS	Actual Wire feed Rate (f) (mm/min)	Deposition rate (d) (mm/min)	Current (i) (Ampere)	Voltage (v) (Volt)	Avg bead width(W) (mm)	Avg bead height (H) (mm)	Aspect ratio (W/H)
1	2900	400	98	12.4	4.72	2.73	1.72
2	3000	500	101	12.6	4.22	1.99	2.12
3	3000	550	99	12.5	4.06	1.87	2.17
4	3000	600	100	12.5	3.97	1.85	2.14
5	3000	650	99	12.4	3.85	1.82	2.11
6	3100	700	98	12.6	3.62	1.76	2.05
7	3100	750	99	12.7	3.46	1.69	2.04
8	3100	800	97	12.7	3.28	1.66	1.97
9	3100	850	96	12.8	3.19	1.64	1.94
10	3100	450	95	12.8	4.21	2.33	1.80
11	2900	350	91	12.4	4.57	2.52	1.81
12	2800	300	88	12.4	5.13	2.61	1.96
13	2800	250	88	12.3	5.28	2.67	1.97
14	2600	200	86	12.1	5.53	3.05	1.81
15	3900	600	120	13.9	4.9	1.9	2.57
16	3900	650	122	13.7	4.71	1.89	2.49
17	4000	700	118	13.7	4.29	1.89	2.26
18	3900	750	120	13.8	4.17	1.87	2.22
19	3900	800	121	13.8	4.12	1.76	2.34
20	3900	850	120	13.9	4.05	1.8	2.25
21	3900	900	121	13.8	3.91	1.61	2.42
22	3900	950	120	13.8	3.91	1.35	2.89
23	3800	550	122	13.6	5.08	2.06	2.46
24	3800	500	121	13.6	5.14	2.21	2.32
25	3800	450	123	13.5	5.58	2.24	2.49
26	3800	400	124	13.6	5.77	2.5	2.30
27	4200	600	144	14.1	5.35	2.29	2.33
28	4100	650	145	14.1	5.19	1.85	2.80
29	4100	700	145	14.2	5.06	1.62	3.12
30	4000	750	144	14.2	4.65	1.68	2.76
31	4100	800	143	14.3	4.66	1.63	2.858
32	4000	850	141	14.1	4.54	1.59	2.855
33	4100	900	141	14.3	4.31	1.43	3.013
34	4200	950	140	14.3	4.25	1.33	3.19
35	4100	1000	141	14.1	4.09	1.31	3.12
36	4100	1050	140	14.2	3.95	1.31	3.01
37	4200	1100	138	14.4	3.96	1.45	2.73
38	4300	550	138	14.2	5.75	2.16	2.66
39	4800	550	160	15.4	5.79	2.06	2.81
40	4800	650	160	15.3	5.81	1.77	3.28
41	4800	750	158	15.3	5.05	1.71	2.95
42	4800	850	157	15.4	4.92	1.73	2.84
43	4800	950	158	15.4	4.63	1.48	3.12
44	4700	1050	155	15.1	4.14	1.4	2.95
45	4800	1150	151	15.4	4.11	1.28	3.21
46	5000	450	159	15.5	6.99	2.23	3.13
47	5800	550	190	16.5	7.36	2.22	3.31
48	5600	750	190	16.4	5.93	1.71	3.46
49	5600	950	189	16.4	5.41	1.57	3.44
50	6300	950	212	16.9	5.83	1.73	3.36

For the aspect ratio study, the wire feed rate (f), deposition rate (d), current (i) and voltage (v) were considered as independent variables (input predictors) because these process parameters were experimentally controlled during the WAAM deposition process. The aspect ratio (AR) was considered as the dependent variable (target response), since it represents the resulting bead geometry influenced by these input parameters. Columns containing average bead height and width data were not taken into consideration.

### Multiple regression

Initially, the multiple regression model was employed to the experimental data to optimise the process parameters of CMT-based wire-arc additively manufactured SS410 walls. Here, the aspect ratio (AR) was considered as the function of four variables namely, wire feed rate (*f*), deposition rate(*d*), current(*i*) and voltage(*v*) which was modelled for the n^th^ experiment by assuming a linear function similar to an earlier study [29].

1 $$AR=\alpha+\beta_1\;f+\beta_2\;d+\beta_3\;i+\beta_4\;v+\varepsilon$$

where AR is the aspect ratio, *f* is the wire feed rate, *d* is the deposition rate, *i* is current and *v* is Voltage.

Equation (1) delineates a linear relationship, where the parameter α signifies the constant or intercept, while *ε *denotes the error associated with the model estimation. The parameters $\:{\beta\:}_{1}$, $\:{\beta\:}_{2}$, $\:{\beta\:}_{3}$ and $\:{\beta\:}_{4}$ represent the anticipated change in the response AR per unit change in variables *f*, *d*, *i* and *v* respectively. This linear model in Eq. (1) assumes that the error *ε* is normally distributed and independent of the variables. Moreover, the four incorporated variables are the most important determinants of aspect ratio in real-world settings.

The multiple regression model result shows that this model can explain 86.55% of variation in the data. The model is as follows (Table 3):

2 $$\begin{aligned}&AR=-2.1288\;+\;\left(-5.8937\times10^{-4}\right)\;f\:+\:3.7571\\&\:\times\:10^{-4}\;d+\left(0.01734929\right)\;i\;+\;\left(0.3241631\right)\;v\end{aligned}$$

**Table 3:** Multiple regression model

Multiple Regression	Coefficients (mean based)	Std. Error
Wire feed Rate (mm/min) [*f*]	-5.8937 × 10^− 4^	0.00028*
Deposition rate (mm/min) [*d*]	3.7571 × 10^− 4^	0.000133**
Current (A) [*i*]	0.01734929	0.00479***
Voltage [*v*]	0.32416316	0.1911
Intercept/constant	-2.1288	1.579
Adjusted_R^2^	**0.8655**	

The standard errors (*=significant at *P* < 0.05, **=significant at *P* < 0.01, ***=significant at *p* < 0.001) indicate that voltage is an insignificant predictor of aspect ratio. Current is the most significant predictor, with a strong positive relationship to the aspect ratio. As the current increases, the aspect ratio increases significantly. Deposition rate is the second most significant predictor; higher deposition rates lead to a higher aspect ratio. Wire feed rate is the third most significant predictor; as the wire feed rate increases, the aspect ratio tends to decrease.

**Fig. 3:**
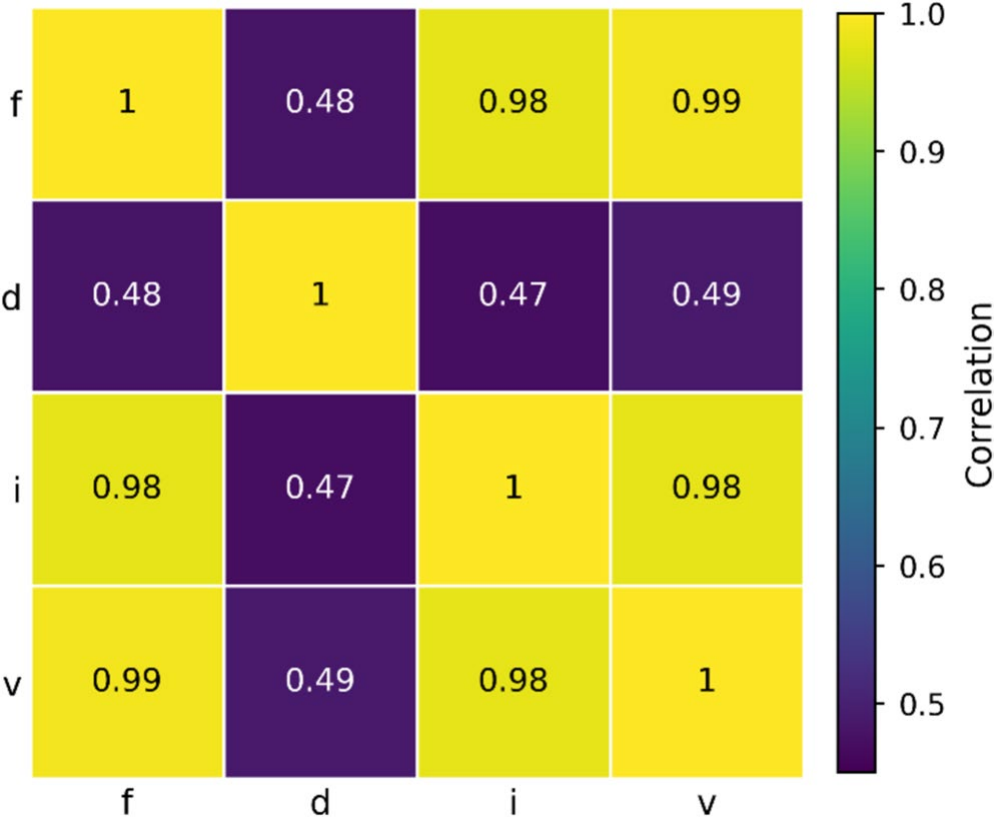
Pearson correlation heatmap depicting the correlation coefficient between two variables

The multiple regression model relies on several assumptions, including linearity as mentioned earlier, absence of multicollinearity and multivariate normality, which entail meticulous scrutinization of the dataset. The correlation plot shown in Fig. 3 indicates strong multicollinearity among several process parameters. In particular, the Pearson correlation coefficients between wire feed rate and current (0.98) and between wire feed rate and voltage (0.99) are extremely high. This strong correlation arises from the synergic control mechanism inherent to the CMT welding system. In such systems, the wire feed rate acts as the primary control parameter, while the welding current and voltage were automatically adjusted by the power source through an internal control algorithm to maintain stable arc conditions and controlled droplet transfer. Consequently, these electrical parameters are physically coupled rather than statistically independent, which naturally results in strong correlations among them in the experimental dataset. Despite this high correlation, none of the variables were removed from the dataset because each parameter represents a physically meaningful process variable that contributes to heat input, arc stability, and material transfer during WAAM deposition. Removing any of these parameters would therefore reduce the physical completeness of the process description. Nevertheless, it should be noted that such multicollinearity may influence the stability and interpretability of coefficient estimates in linear regression models. As a result, multiple regression model cannot fully capture the complexity inherent in the dataset, constrained by its underlying assumptions and can explain only 86.55% variation in our experimental data.

Considering the limitations associated with multiple regression model, various sophisticated tree-based regression models such as Random Forest Regressor (RFR), XGBoost (XGB), Extra Tree Regressor (ETR), Cat-Boost Regressor (CBR) were explored to capture the intricacies present in the experimental data. These tree-based ensemble methods were selected because they are well suited for WAAM modelling. They can capture complex nonlinear interactions among process parameters, handle correlated inputs arising from the synergic control of the CMT system and provide robust predictive performance even with relatively small experimental datasets. These characteristics make them attractive tools for modelling process–geometry relationships in WAAM, where the underlying physical phenomena are highly coupled and difficult to represent using predefined mathematical functions.

The experimental database (refer to Table 2) was divided into training and testing subsets using an 80:20 split, where 80% of the data was used to train the models and the remaining 20% was reserved as an independent test set to evaluate the predictive performance of the models. To further assess the robustness of the models and reduce the risk of overfitting, five-fold cross-validation was also performed using the full dataset.

All of the models mentioned above were trained using four predictors namely, wire feed rate (*f*), deposition rate (*d*), current (*i*) and voltage (*v*) with the target variable as aspect ratio. These models also require two key parameters, the number of trees (t) and the maximum depth (m) where maximum (max) depth is the largest depth of each tree. The randomness within the model stems from the random sampling of data and features and it enables these models to effectively capture the complexity of dataset. To explore the sensitivity of predictions to the number of trees and the maximum depth, randomized grid search and Bayesian optimisation methods were used for tuning the hyperparameters of these regression models. While grid search exhaustively searches all possible combination of selected hypermeters and is computationally expensive and time-consuming with wide range of hyperparameters [30, 31], random search is more efficient as it randomly samples a fixed number of hyperparameters. However, random search does not guarantee the best optimised hyperparameters, as it can miss good combinations. Bayesian optimisation can often quickly find good hyperparameter settings, as it uses a probabilistic model to balance exploration and exploitation of the hyperparameter space [32].

The performance or goodness-of-fit of these regression models was evaluated using the pseudo_R^2^ metric, which relates the Out-Of-Bag (OOB) mean squared error obtained from the ensemble models to the statistical variance of the target variable (aspect ratio) [29]. The variance of the target variable is defined as:

3 $$var\left(y\right)\:=\:\frac1n\:\sum\:_{i=1}^n{(y_i-\overset-y)}^2$$

where $\:{\mathrm{y}}_{i}$ represents the experimentally measured aspect ratio for the *i*^*th*^ observation, $\:\stackrel{-}{y}\text{}$ denotes the mean aspect ratio of the dataset, and *n* is the total number of experimental samples. Using this definition, the pseudo_R² metric was computed as:

4 $$\:pseudo\_R^2=1-\frac{{MSE}_{OOB}}{var\left(y\right)}$$

where MSE_OOB_ represents the Out-Of-Bag mean squared error estimated from the ensemble learning models. This metric indicates the proportion of variance in the target variable that is explained by the model predictions.

### Random forest regressor (RFR)

Random forest belongs to the ensemble learning family that combines multiple decision trees. Each decision tree is constructed based on a bootstrapped sample of the original dataset (i.e., a random sample of data points with replacement). At each split in the tree, a random subset of features is considered when determining the best split. The final prediction is typically the average (mean) of the predictions made by each tree in the forest.

Hyperparameter-tuning was performed by setting the number of trees (t) in the range of 10 to 1000, and max_depth (m) in the range of 1 to 10. Random search identified t = 1000, and m = 6 as the optimal hyperparameters, while Bayesian optimization suggested t = 800 and m = 6 as the best hyperparameter settings. Additionally, the performance of RFR model was evaluated for various values of t = (300, 500, 800, and 1000) and m = (3, 4, 5, and 6) to compare and ensure the attainment of the best model. The importance scores of four regressors across twenty sets of regressions is presented in Table 4. The best fit estimation obtained at optimal hyperparameters (t = 800, m = 6) shows that voltage is the best predictor of aspect ratio, followed by current.

**Table 4:** Importance score for various trees and max depth for RFR model (seed=99)

	t = 300				t = 500				t = 800			
m	6	5	4	3	6	5	4	3	6	5	4	3
Wire feed Rate [f]	0.02926	0.02460	0.0101	0.0070	0.029564	0.02563	0.01022	0.00694	0.03047	0.02550	0.00954	0.0073
Deposition rate [d]	0.00297	0.00215	0.0060	0.0067	0.003990	0.002386	0.00560	0.00705	0.00308	0.00254	0.00602	0.0068
Current [i]	0.1056	0.09139	0.0452	0.0424	0.10663	0.08998	0.04404	0.04136	0.10526	0.09001	0.04447	0.0417
Voltage [v]	0.1158	0.0956	0.0430	0.0482	0.11579	0.104261	0.04195	0.04943	0.12009	0.09935	0.04186	0.0477
Variation (var)	85.1524	85.1635	85.084	84.778	85.1773	0.03664	85.1063	84.8105	85.288	85.280	85.214	84.91
MSE (OOB)	0.03672	0.03669	0.0368	0.0376	0.03666	0.03664	0.03681	0.03753	0.03636	0.03638	0.03653	0.0372
Pseudo R^2^	0.9568	0.9569	0.9566	0.9556	0.9569	0.9569	0.9567	0.9557	0.9573	0.9573	0.9571	0.9561
t = 1000												
m	6	5	4	3								
Wire feed Rate [f]	0.029935	0.0250494	0.00967	0.006763								
Deposition rate [d]	0.003022	0.0028512	0.0062309	0.0072564								
Current [i]	0.106963	0.087693	0.0447707	0.0410155								
Voltage [v]	0.119243	0.0998595	0.042236	0.0482626								
Variation (var)	85.26155	85.2606	85.20147	84.899								
MSE (OOB)	0.036424	0.036422	0.036553	0.037284								
Pseudo R^2^	0.9572	0.95728	0.95709	0.95608								

### Extreme gradient boosting (XGBoost)

Extreme gradient boosting (XGBoost) is an ensemble of decision trees that builds trees (weak learners) sequentially. Each tree focuses on correcting the errors made by the previous ensemble.

Bayesian optimisation suggested the optimised hyperparameters of t = 300 and m = 3, while randomised grid search identified t = 300, and m = 4 as the optimal hyperparameter settings. Like the RFR model, the performance of XGB model was assessed for additional values of t = (500, 800) and m = (3,4) to avoid any oversight and ensure the best possible model.

The importance scores of three predictors across six sets of regressions is presented in Table 5. The importance score for m = 2, 5 and 6, are omitted from Table 5, due to their poor performance. The best fit estimation obtained at optimal hyperparameters (t = 300, m = 3) shows that current (*i*) is the best predictor of aspect ratio. This contrasts with the RFR model, where voltage was found to be the most significant predictor. This difference suggests that the XGB model may capture different relationships in the data compared to the RFR model.

**Table 5:** Importance score for various trees and max depth for XGB model (seed=99)

	t=300		t=500		t=800	
m	4	3	4	3	4	3
Wire feed Rate	0.06121	0.05718	0.04328	0.05362	0.06259	0.05540
Deposition rate [d]	0.02364	0.03345	0.02354	0.02137	0.027527	0.03067
Current [i]	0.07140	0.11174	0.07935	0.07536	0.068447	0.08021
Voltage	0.01880	0.01734	0.01913	0.01663	0.024509	0.01295
Variation (var)	83.1608	83.7589	83.0967	83.6835	83.2067	83.7068
Pseudo R^2^	0.9500	0.9522	0.9498	0.95194	0.95025	0.95204

### Cat-boost regressor (CBR)

CBR is another gradient boosting algorithm similar to XGBoost. It uses gradient boosting over decision trees to minimise the error between predicted and actual target values. CBR efficiently handles categorical features, eliminating the need for manual pre-processing.

The optimal hyperparameters observed from Bayesian optimisation technique were t = 800 and m = 2, while randomised grid search suggested optimal settings of t = 500 and m = 2. The CBR model was further executed for the varying values of t = (300, 500, 800) and m = (1, 2, 3), like earlier models. The importance scores of four predictors across nine sets of regressions are presented in Table 6. The best fit estimation obtained at optimal hyperparameters of (t = 800, m = 2), shows that current is the best predictor of aspect ratio followed by voltage, deposition rate and wire feed rate. This indicates that the current has most significant impact on the aspect ratio in our dataset. CBR shows the highest variation of 85.688 with the highest Pseudo R^2^ score of 0.95888 among all regression models; thus, CBR can be considered as the best model for this dataset.

**Table 6:** Importance score for various trees and max depth for CBR model (seed=99)

	t = 300			t = 500			t = 800		
m	3	2	1	3	2	1	3	2	1
Wire feed Rate [f]	0.02672	0.01810	0.02044	0.01666	0.01893	0.02011	0.02386	0.01535	0.01911
Deposition rate [d]	0.01962	0.02695	0.01445	0.03696	0.0330	0.01656	0.03179	0.015704	0.01882
Current (A) [i]	0.09222	0.10005	0.08349	0.09805	0.07131	0.09142	0.092815	0.07939	0.1098
Voltage [v]	0.05398	0.03931	0.05272	0.04425	0.05767	0.02346	0.045826	0.04967	0.03471
Variation (var)	85.5605	85.6126	85.3425	85.5715	85.6159	85.4041	85.6657	85.6881	85.452
Pseudo R^2^	0.9584	0.9586	0.9577	0.9584	0.9586	0.9579	0.95881	0.95888	0.9581

Moreover, theoretically, it is widely understood that electrical current directly influences the aspect ratio by affecting the heat generation. Higher welding current increases arc energy and heat input, leading to greater melting and consequently influencing bead geometry and aspect ratio [33]. Conversely, voltage indirectly influences the aspect ratio by affecting the shape and stability of the arc. Additionally, it has been suggested [34] that the bead width can be significantly influenced by the voltage and travel speed, whereas the bead height is largely affected by the welding current and travel speed. Thus, the observations made from the CBR regression model align with the theoretical expectations and reinforce the validity of our findings.

It is worth noting that different machine learning algorithms may produce slightly different feature importance rankings because of differences in their internal learning mechanisms. RFR determines feature importance based on the reduction of impurity across decision tree splits, whereas boosting-based algorithms such as XGBoost and Cat-Boost sequentially optimise prediction errors during training. In datasets where several predictors are strongly correlated, as observed in the present CMT welding system, these algorithms may distribute predictive importance differently among correlated features. From a physical standpoint, the importance obtained from the Cat-Boost model aligns more closely with the established understanding of welding processes. In arc-based additive manufacturing, the welding current directly governs the heat input supplied to the molten pool, which controls melting behavior, penetration depth, and material transfer rate. Consequently, variations in current have a strong influence on bead height and the resulting aspect ratio. On the other hand, welding voltage primarily controls arc length and arc stability, which affects the spreading of molten metal and therefore influences bead width. The differences observed between the Random Forest and boosting-based models may therefore arise from the redistribution of importance among correlated electrical parameters within the synergic CMT system.

## Comparative analysis

In this section, we compare the results of Multiple Regression, RFR, XGBoost, and Cat-Boost Regression to evaluate their effectiveness in predicting the value of aspect ratio during WAAM. The Extra Trees Regressor (ETR) model was also evaluated; however, as its performance did not significantly exceed that of the Random Forest model, its detailed results are provided as the Supplementary Material.

**Fig. 4:**
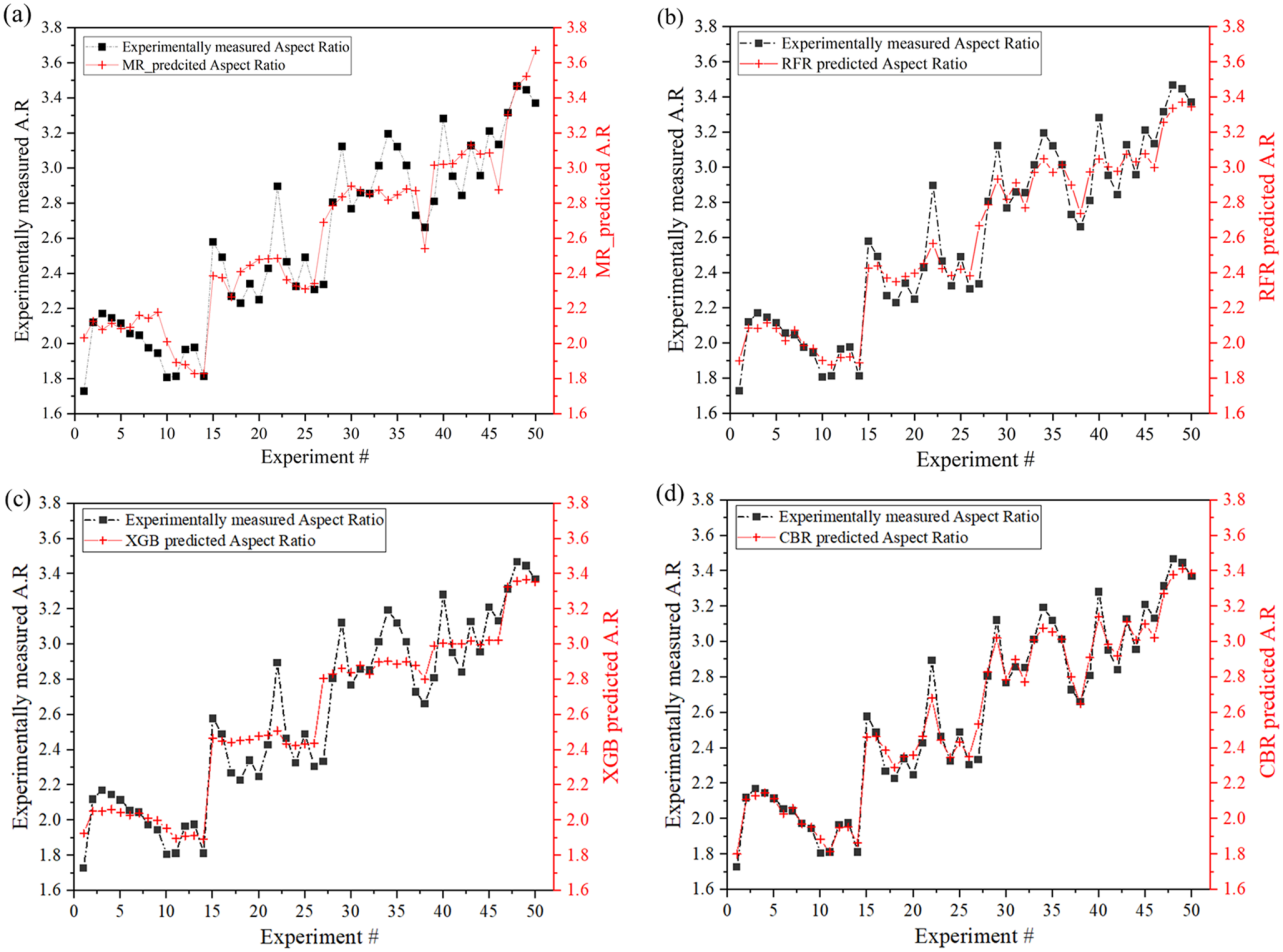
Comparison of experimentally observed aspect ratio values with predictions from (**a**) MR, (**b**) RFR, (**c**) XGB, (**d**) CBR models

**Fig. 5:**
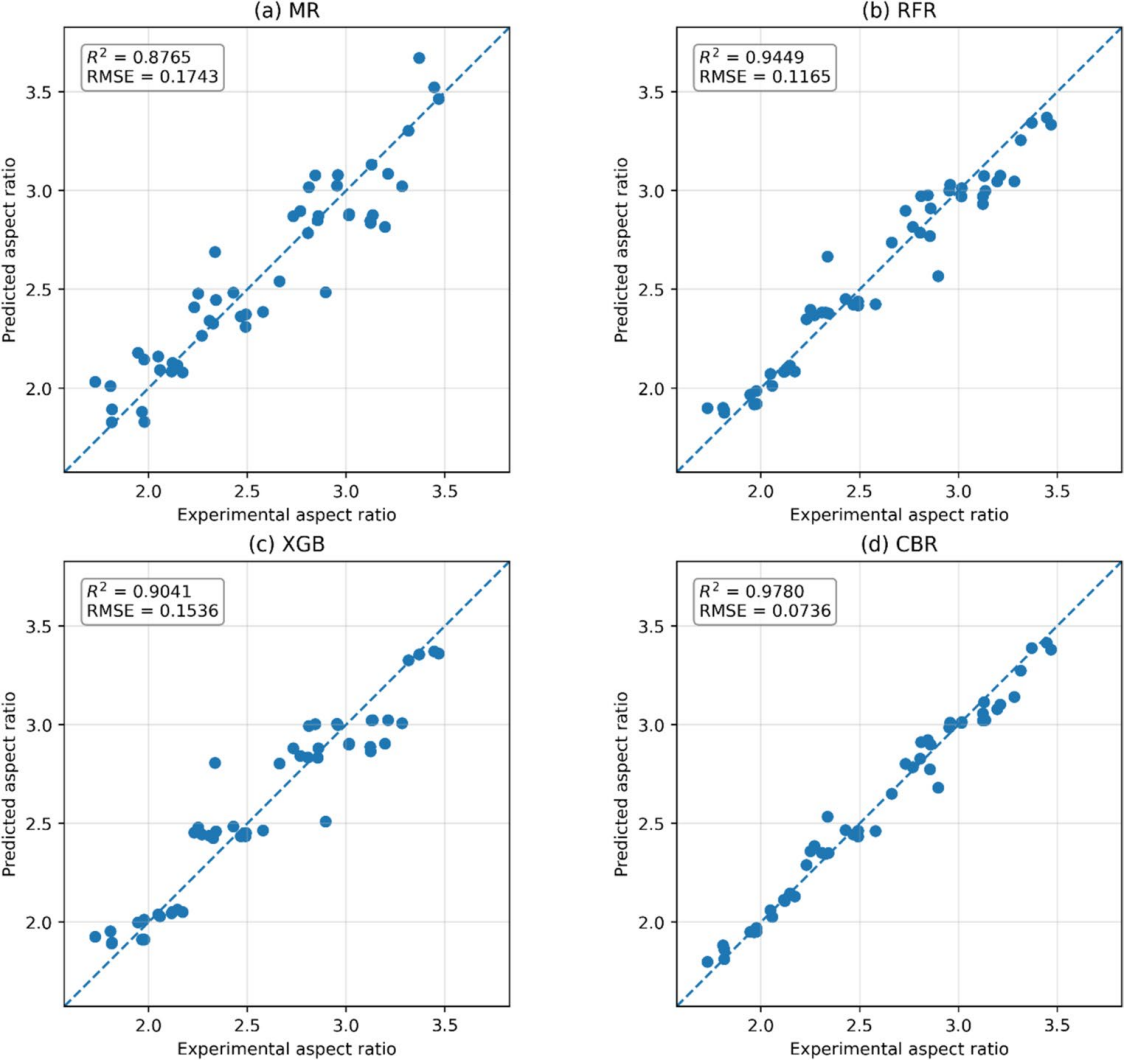
Parity plots comparing experimentally measured and predicted aspect ratio values for (**a**) Multiple Regression (MR), (**b**) Random Forest Regressor (RFR), (**c**) XGBoost (XGB) and (**d**) Cat-Boost Regressor (CBR). The dashed line represents the ideal parity line $\:y=x$, indicating perfect agreement between experimental and predicted values

Figure 4(a–d) compares the experimentally measured aspect ratio values with the predictions obtained from the four regression models. To further illustrate the predictive accuracy of the regression models, parity plots comparing the experimentally measured and predicted aspect ratio values are presented in Fig. 5. In Fig. 5, the dashed line represents the ideal parity line $\:\left(y=x\right)$, corresponding to perfect agreement between predicted and experimental values. It can be observed that the predictions from the Cat-Boost Regressor (CBR) are more closely aligned with the parity line compared with the other models, indicating superior predictive performance. The Random Forest and XGBoost models also demonstrate good agreement with experimental measurements, whereas the multiple regression model shows comparatively larger deviations.

From Fig. 4(a)-(d), it is evident that all the tree-based models outperformed the multiple-regression model in predicting the aspect ratio. Although all tree-based models demonstrated better accuracy when the aspect ratio was below 2.2, the CBR model maintained superior performance even for higher aspect ratio. Overall, the CBR model showed a more consistent trend in its predictions compared to other tree-based models.

Table 7 presents the standard deviations of the differences between the predicted values from the three tree-based models (excluding multiple regression) and the actual values across the entire set of experiments. The CBR model exhibited the lowest standard deviation, indicating that it is a better model for WAAM compared to the other tree-based regression models (RFR and XGB). To provide a more comprehensive assessment of predictive accuracy, standard regression error metrics, including Root Mean Square Error (RMSE) and Mean Absolute Error (MAE) were calculated using the independent test dataset. The comparative results for all models are summarized in Table 8. In addition, Table 9 presents the five-fold cross-validation results obtained using the full experimental dataset for all employed ML models. This indicates that the CBR model provides the most reliable predictive performance among the evaluated models for the present WAAM dataset.

**Table 7:** Standard deviation of three models for whole set of experiment

	RFR	XGB	CBR
Standard deviation of residuals (experimental values vs. predicted values for the whole experiment)	0.1174	0.1552	0.0742

**Table 8:** Performance comparison of ensemble-learning models using independent test-set evaluation metrics

Models	*R*^2^_score	MAE	RMSE
RFR	0.6981	0.2375	0.2852
XGB	0.6575	0.2454	0.3037
CBR	0.7068	0.2349	0.2810

**Table 9:** Five-fold cross-validation score

Models	5-fold CV score
RFR	0.7535
XGB	0.7522
CBR	0.7589

The experimental investigation was further extended to analyze the microstructural behaviour and mechanical properties of thick walls produced under optimal fabrication parameters (processing parameters resulting in aspect ratio of 3.4678). This included a detailed evaluation of the material’s mechanical performance and an in-depth examination of the microstructure as a function of build height, aiming to assess how the fabrication conditions influenced the structural integrity and properties of the thick walls.

## Microstructural analysis

**Fig. 6:**
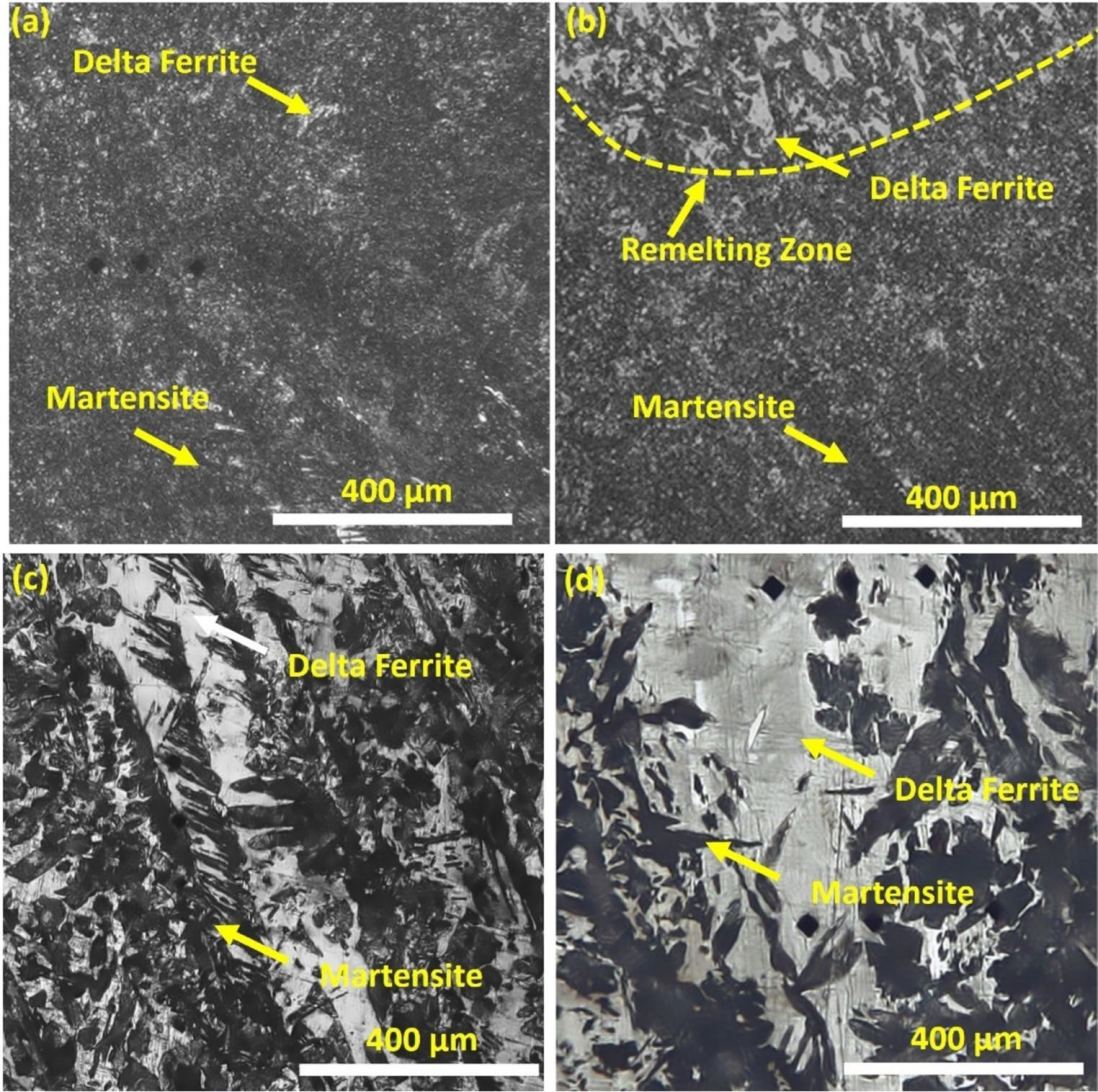
The optical micrographs of SS410 thick wall built at optimum parameters as a function of build height from bottom to top (**a**-**d**)

**Fig. 7:**
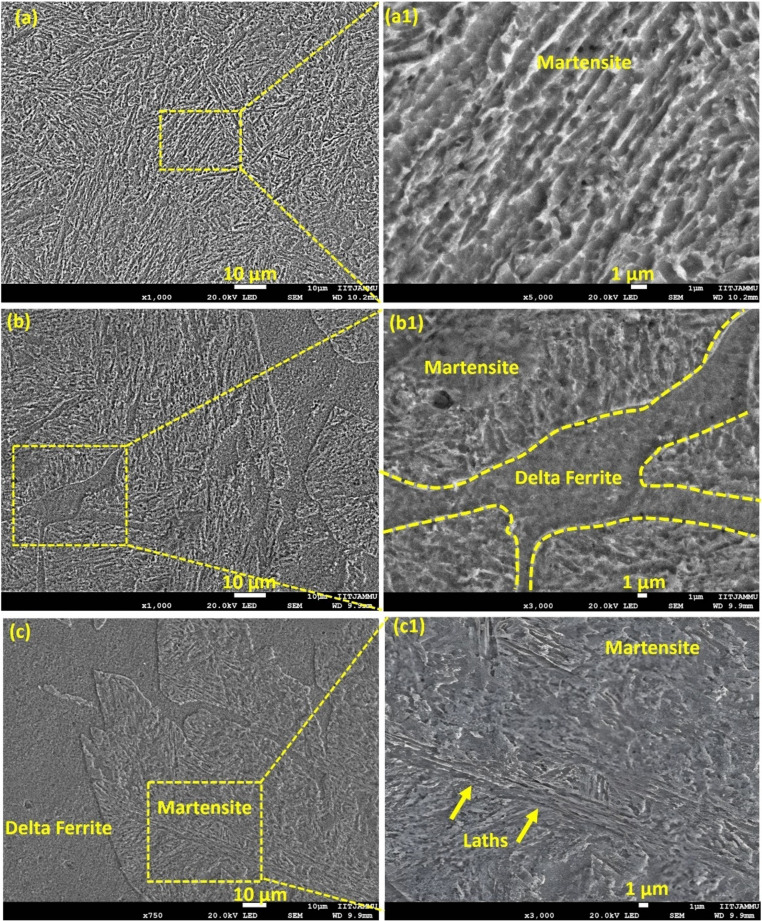
Scanning electron microscope images of the thick wall at varying build heights along with its magnified view: (**a**, a1) bottom, (**b**, b1) middle, and (**c**, c1) top

Figure 6 shows the microstructural images captured by the optical microscope at several locations as a function of build height. The images clearly demonstrate the heterogeneities that occurred during the bulk production process, as the microstructure evolves with build height. It is important to mention that the phase at the bottom region exhibits an abundance of martensite (Fig. 6(a)), which may be related to the rapid cooling rate. The findings obtained using optical methods were seen consistent with the images obtained from the scanning electron microscopy, as shown in Fig. 7(a). Furthermore, an increase in the build height resulted in a decrease in the presence of the martensitic phase. The luminous island-like characteristics appear inside the remelting zone of the martensite matrix (Fig. 6(b)). This observation indicates that the presence of delta ferrite is enhanced by the remelting process, with delta ferrite exhibiting island-like features that extend throughout the depth of penetration. The SEM image shows a type of morphology skeleton for the delta ferrite (Fig. 7(b)).

As the build height increased, the remelting zone became less distinctly identifiable; however, there was a notable and significant increase in the fraction of delta ferrite (Fig. 6(c), (d)). The higher concentration of delta ferrite in the overlap area compared to the build height is caused by additional melting, which creates a larger zone of re-melting that extends into the neighbouring beads. This allows for greater separation of ferrite stabilizers during the solidification process. By implementing proper segregation, the development of delta ferrite can remain stable throughout the phase transition process [35]. The morphology of the delta ferrite changes as the build advances. The SEM image of the top sample confirms the existence of the coarser and blocky type delta ferrite (Fig. 7(c)). Previous studies have also demonstrated a variation in the physical characteristics of delta ferrite with cooling rate [36].

**Fig. 8:**
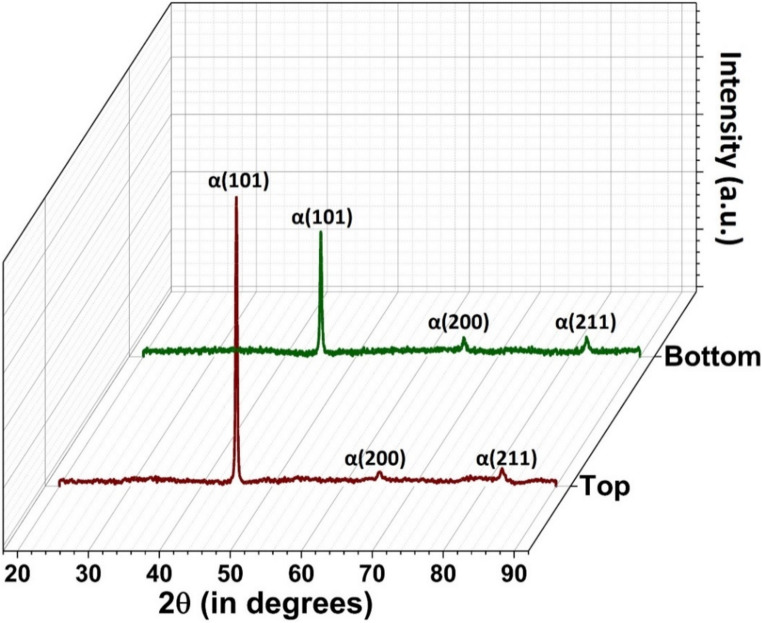
The XRD diffractograms of the thick wall at two specific positions designated as bottom and top

To further investigate the possible presence of austenite, X-ray diffraction was used and the results are shown in Fig. 8. The XRD peaks indicated that the dominant phases present in the deposited material was ferrite and martensite, with no detectable retained austenite within the detection limits of the measurement. The results indicated that the predominant phases present are either body-centered cubic ferrite or martensite.

Table 10 highlights the mechanical properties of the samples, determined based on their construction height. In contrast to the bottom specimens, the results show a reduction in the ultimate tensile strength (UTS) and yield strength (YS) values for the top samples. In addition, the top samples exhibit the most notable standard deviation in UTS and YS, which may be attributable to the significant presence of delta ferrite in these locations. This observation aligns well with the research conducted by Roy et al. [37] that examines the relationships between heterogeneities and delta ferrite content. The bottom section of the specimen used in the present investigation is defined as the area that extends up to a height of 12 mm. The hardness near the bottom exhibits the largest value and a substantial standard deviation. This accounts for the pronounced disparity in hardness between the martensite phase and the delta ferrite phase. In contrast, the top regions exhibit relatively lower hardness values with a smaller standard deviation, indicating a reduced difference between the two distinct phases.

**Table 10:** The mechanical properties and density results of bottom and top samples fabricated at optimum parameters using wire arc additive manufacturing process

Sample	Properties				
	UTS(MPa)	YS(MPa)	Hardness(HV)	PercentageElongation (%)	Density(g/cc)
Bottom	765 ± 39	645 ± 19	329 ± 50	2.25 ± 1.49	7.7075 ± 0.003
Top	655 ± 60	563 ± 36	245 ± 28	1.43 ± 0.02	7.688 ± 0.02

This behaviour can be attributed to the progressive reduction in cooling rate caused by heat accumulation during multilayer deposition. The highest hardness of 452 HV could be attributed to the highest cooling rate resulting in fine lath structures [38]. As the build progresses, the cooling rate diminishes due to the rise in interlayer temperature, which ultimately leads to slower cooling [2]. As a consequence, the hardness of the martensite in the top regions decreases due to the development of coarse lath sizes at slower cooling rates [39]. Besides, the property evaluation signifies poor percentage elongation in the top sample, which can be ascribed to the large delta ferrite contents that usually promote brittle failure. Analysis of the density values revealed no substantial difference. As a result, the porosity remains consistent throughout all samples. Furthermore, it is important to highlight that the density attained in the current study is similar to that of the wrought counterpart [40] indicating that the AM process can produce parts with comparable density and potentially similar mechanical integrity as wrought materials.

**Fig. 9:**
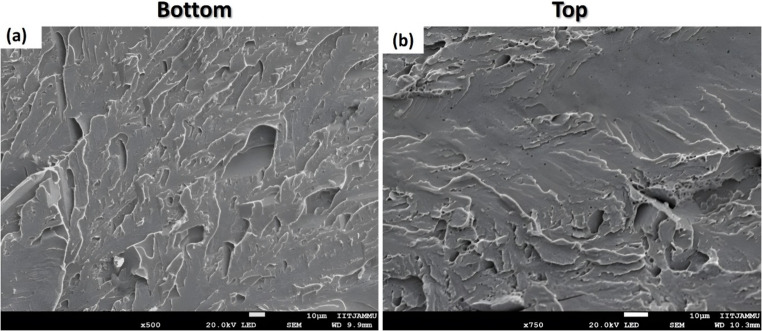
Fractographic images of the tensile-tested specimens: (**a**) bottom and (**b**) top, depicting the fracture surfaces

The fractography images shown in Fig. 9 illustrate the specific kind of fracture that occurred during mechanical testing. Evidence of brittle fracture was evidenced from the cleavage facets depicted in Figs. 9(a, b). It should be noted that the cleavage facet formation occurs in a plane perpendicular to the applied tensile stress [41]. Based on the research reported in literature [42], it was inferred that the brittle fracture occurs when cracks initiate from brittle precipitates within the grain boundaries.

## Conclusion

This study presents a machine-learning-based modelling framework for predicting bead geometry during CMT-based WAAM fabrication of SS410 stainless steel. A new experimental database of 50 experimentally developed beads was prepared and utilized to predict the part aspect ratio. In this study, advanced tree-based regression models such as Random Forest, Extra Trees Regressor, XGBoost and Cat-Boost Regressor were applied to model bead geometry in WAAM-processed SS410. The Cat-Boost Regression model exhibited an excellent correlation with the results revealing a standard deviation of 0.042. The best aspect ratio achieved was 3.46, corresponding to a wire feed rate of 5600 mm/min, a deposition rate of 750 mm/min, electrical current of 190 amperes and a voltage of 16.4 volts.

Based on the extensive models developed in this study, several key conclusions are derived:

1. A novel modelling approach utilizing advanced tree-based regression models was applied to the CMT-based WAAM process for the first time. These models demonstrated higher accuracy than that the multiple regression model with linear assumptions. Although, all tree-based models demonstrated greater accuracy when the aspect ratio was below 2.2, the CBR maintained the superior performance even for the higher aspect ratio values, exhibiting a more consistent trend in its predictions compared to other tree-based models.
2. Similar to other deposition processes, the experimental results indicated that the aspect ratio during CMT based WAAM is most significantly influenced by the electrical current followed by the voltage. The CBR model also revealed a similar trend, aligning with existing literature, where current directly influences the aspect ratio by affecting heat generation, while voltage indirectly influences the aspect ratio by affecting arc shape and stability. Moreover, numerous studies highlight that current is crucial for maintaining bead height, while voltage is essential for bead width. Although current plays a dominant role compared to voltage, both are crucial for achieving closely packed and crack-free thicker walls.
3. The multiple regression model equation was found to explain 86.55% of the variation in predictions of aspect ratio compared to the experimental results.
  $$\begin{aligned}&AR=-2.1288\;+\;\left(-5.8937\:\times\:10^{-4}\right)\;f\:+\\&\:3.7571\:\times\:10^{-4}\;d\;+\;\left(0.01734929\right)\;i\;+\;\left(0.32416316\right)\;v\end{aligned}$$

where AR is aspect ratio, *f* is wire feed rate, *d* is deposition rate, *i* is current and *v* is the voltage.

4. The microstructural analysis and mechanical properties analysed of the processed optimal part demonstrated significant heterogeneity in microstructural phases along the build height. The results indicate a progressive reduction in martensite and a corresponding increase in delta ferrite as the build advances. This may be attributed to the reduction in the cooling rate that favours delta ferrite formation. Compared to the top regions, the bottom sample exhibits 16.8%, 14.6% and 34.3% enhancement in the ultimate tensile strength, yield strength and hardness values, respectively. This could be ascribed to the relatively low fraction of the detrimental phase (delta ferrite) in the bottom sections.

## Supplementary information

Below is the link to the electronic supplementary material.

Supplementary File 1 (DOCX 65.1 KB)

## Abbreviations

AM: Additive manufacturing
ANN: Artificial neural networks
AR: Aspect ratio
BH: Bead height
BW: Bead width
CBR: Cat-Boost Regression
CFD: Computational fluid dynamics
CMT: Cold metal transfer
i: Current (Ampere)
d: Deposition rate (mm/min)
ETR: Extra Tree Regressor
FEM: Finite element method
h-TLBO: Hybrid-teaching learning-based optimization
m: Max_depth
ML: Machine learning
MSE: Mean squared error
t: Number of trees /n_estimators
OOB: Out-Of-Bag
RFR: Random Forest Regressor
RSM: Response surface methodology
TS: Travel speed (deposition rate)
UTS: Ultimate tensile strength
v: Voltage (V)
WAAM: Wire-arc additive manufacturing
WFR/WFS: Wire feed rate/speed (mm/min)
XGB: XGBoost
YS: Yield strength
