# Supplementary material for: Machine learning informed additive manufacturing of stainless steel 410 using cold metal transfer-based metal inert gas welding
Source: Int J Adv Manuf Technol. 2026 Apr 18;144(5-6):3875–92. doi: 10.1007/s00170-026-18086-6 (PMC13260246; doi:10.1007/s00170-026-18086-6)

**Extra Tree Regressor (ETR)**

ETR is similar to random forest, in that it builds an ensemble of trees using the bootstrap aggregating (bagging) technique. Moreover, ETR introduces additional randomness by selecting a random subset of features at each split within a decision tree. ETR model was executed for the varying values of t = (300, 500, 800) and m = (2, 3, 4). The max_depth (m) was not increased beyond m=4, as no improvement in the variation explained by the model was observed for increasing number of trees i.e., m = 5 and 6. The importance scores of three regressors across nine sets of regressions is presented in Table 5. The best fit estimation (t=800, m= 3) shows that current is the best predictor of aspect ratio, followed by voltage.

**Table 1s.** Importance score for various trees and max depth for ETR model (seed=99).

|  | **t = 300** | | | **t = 500** | | | **t = 800** | | |
| --- | --- | --- | --- | --- | --- | --- | --- | --- | --- |
| **m** | **4** | **3** | **2** | **4** | **3** | **2** | **4** | **3** | **2** |
| Actual Wire feed Rate (mm/min) | 0.019625 | 0.01568 | 0.034609 | 0.02046 | 0.01559 | 0.034717 | 0.01972 | 0.0152 | 0.03494 |
| Deposition rate (mm/min) [d] | 0.002517 | 0.000792 | -0.00141 | 0.002753 | 0.001099 | -0.001277 | 0.002621 | 0.000799 | -0.00135 |
| Current (A) [i] | 0.039404 | 0.04699 | 0.06618 | 0.03871 | 0.048393 | 0.066715 | 0.03870 | 0.04856 | 0.0659 |
| Voltage | 0.02993 | 0.030303 | 0.04893 | 0.03099 | 0.030032 | 0.04885 | 0.03002 | 0.030555 | 0.048847 |
| Variation (var) | 84.9582 | 85.1852 | 82.7037 | 85.1187 | 85.3341 | 82.7885 | 85.14461 | **85.34579** | 82.78679 |
| MSE(OOB) | 0.03701 | 0.03645 | 0.042575 | 0.03661 | 0.03609 | 0.0423669 | 0.03655 | 0.03606 | 0.042368 |
| Pseudo R^2^ | 0.9564 | 0.9572 | 0.94852 | 0.9569 | **0.9577** | 0.94882 | 0.95707 | **0.95774** | 0.94882 |

**Table 2s.** Performance assessment of ETR model.

| **Models** | **R^2^_score** | **MAE** | **RMSE** | **5-fold CV score** |
| --- | --- | --- | --- | --- |
| ETR | 0.6753 | 0.2509 | 0.2957 | 0.7514 |

|  | **ETR** |
| --- | --- |
| Standard deviation of experimental values vs. predicted values for the whole experiment | 0.0979 |


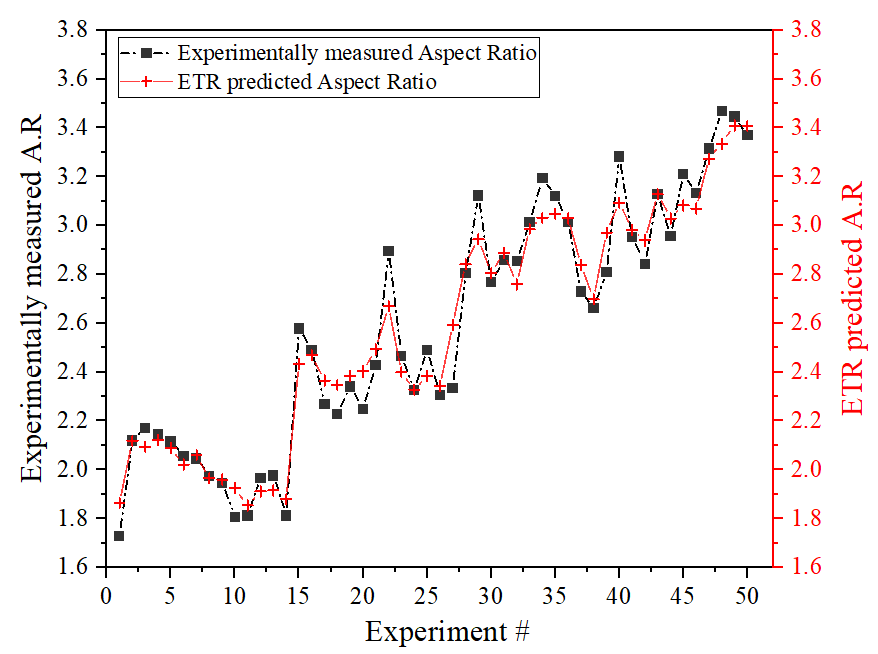

Supplement: Supplementary file 1 — (DOCX 65.1 KB) [file 170_2026_18086_MOESM1_ESM.docx]
